# Left Right Patterning, Evolution and Cardiac Development

**DOI:** 10.3390/jcdd1010052

**Published:** 2014-04-08

**Authors:** Iain M. Dykes

**Affiliations:** Department of Cardiovascular Medicine and Wellcome Trust Centre for Human Genetics, University of Oxford, Roosevelt Drive, Headington, Oxford, OX3 7BN, UK

**Keywords:** left-right patterning, heterotaxy, isomerism, Nodal, Pitx2c

## Abstract

Many aspects of heart development are determined by the left right axis and as a result several congenital diseases have their origins in aberrant left-right patterning. Establishment of this axis occurs early in embryogenesis before formation of the linear heart tube yet impacts upon much later morphogenetic events. In this review I discuss the differing mechanisms by which left-right polarity is achieved in the mouse and chick embryos and comment on the evolution of this system. I then discus three major classes of cardiovascular defect associated with aberrant left-right patterning seen in mouse mutants and human disease. I describe phenotypes associated with the determination of atrial identity and venous connections, looping morphogenesis of the heart tube and finally the asymmetric remodelling of the embryonic branchial arch arterial system to form the leftward looped arch of aorta and associated great arteries. Where appropriate, I consider left right patterning defects from an evolutionary perspective, demonstrating how developmental processes have been modified in species over time and illustrating how comparative embryology can aide in our understanding of congenital heart disease.

## 1 Introduction

The symmetrical appearance of the body is superficial and belies a highly asymmetrical arrangement of the viscera. Unpaired organs such as the stomach and spleen are displaced to one side of the body while paired structures such as the lungs exhibit an asymmetric morphology. Heterotaxy syndrome is a spectrum of diseases in which abnormal left-right positioning and morphology of these organs occurs [1–3]. Many congenital heart diseases have their origins in aberrant left-right patterning (LRP) illustrating the importance of sidedness for many aspects of cardiovascular development.

Early in its development, the vertebrate embryo is bilaterally symmetrical and this is reflected in the development of the heart which is formed from paired heart fields that come together to form a linear heart tube [4]. Later development is characterised by asymmetry first manifesting in the looping morphogenesis of the heart tube [5] and later in the divergent differentiation of the atria and ventricles and in the asymmetric remodelling of the arterial and venous connections [6,7].

In this review I will consider how the left-right axis controls many aspects of heart development. I will first discuss the genetic pathways that specify the axis early in embryogenesis before going on to discuss cardiac phenotypes associated with defective patterning. I will demonstrate how an understanding of the evolution of these processes can aide in the interpretation of congenital diseases.

## 2 Establishment of Left-Right Asymmetry in the Early Embryo

The embryo has evolved a finely tuned system to tell left from right and this axis is established in the mesoderm before formation of the linear heart tube. Right-sidedness represents the baseline state in all known species and in the absence of instruction to do otherwise both sides of the body will develop according to a right-sided plan, a state known as right isomerism (discussed below). Left sidedness must be induced, leading to expression of a Left-specific programme of gene regulation which directs differentiation away from the default state on one side of the body.

The establishment of the left-right axis may be viewed as a multi-step process. The first stage is an act of symmetry breaking, the creation of a transient difference between the two formerly equivalent sides, and most researchers agree that a transient, midline organiser region called the node is central to this process in all embryos, although the mechanism by which this is achieved may be quite different. This transient difference is then perceived and translated into differential gene expression in perinodal cells. Next, the signal is relayed to the lateral plate mesoderm in which a second phase of differential gene expression is initiated. Lateral plate mesoderm derived cells contribute to many visceral organs including the heart and positional information derived from the left-right axis determines many aspects of their development. I will begin this review by describing how this process occurs in the mouse embryo, which is perhaps the best understood, before contrasting this with the quite different mechanisms operating in the chick embryo. These two embryos seem to represent the two major modes of symmetry breaking seen in the animal kingdom. I will here provide a brief overview of the process but for a more detailed discussion the reader is referred to reviews by Chen *et al.* [8] and Hamada *et al.* [9].

### 2.1 Axis Establishment in the Mouse Embryo

**Figure 1:**
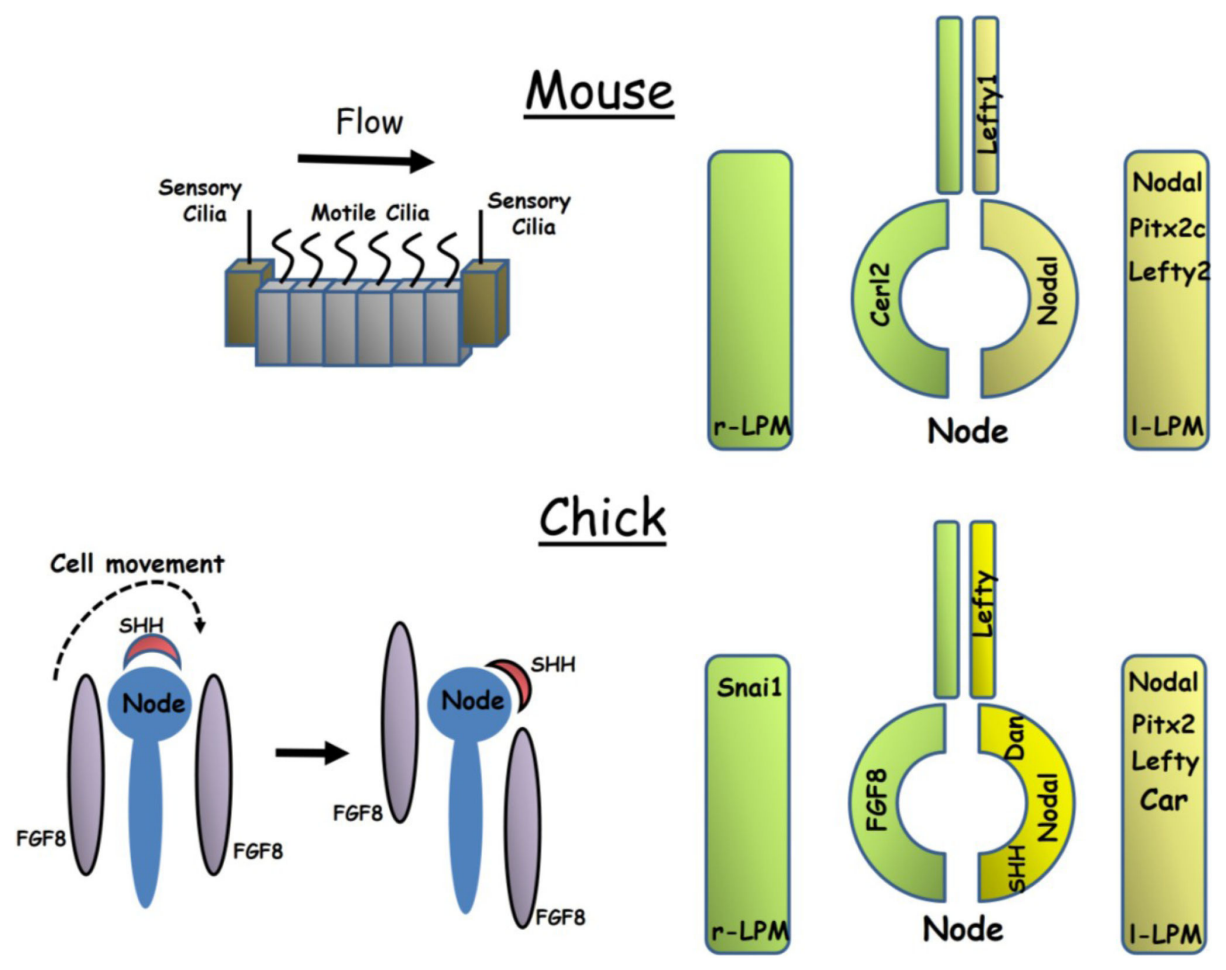
The mouse and chick embryos represent the two major mechanisms of symmetry breaking seen in vertebrates. In the mouse (**top left**), a leftward fluid flow is generated across the node by rotational beating of motile cilia while in the chick embryo (**lower left**) a leftward movement of cells transforms the initially symmetrical expression of *Shh* and *Fgf8* into an asymmetric one. The diagrams on the right illustrate similarities and differences between the two embryos in the expression of key genes within the node, lateral plate mesoderm (LPM) and midline floorplate. Note that many additional genes not shown are also asymmetrically expressed in chick.

Symmetry breaking in the mouse occurs at E7.75. The node of the mouse resembles a fluid filled pit in which current is generated by means of motile cilia borne by the nodal pit cells (Figure 1). These cilia have a tilted morphology and are oriented towards the posterior. This means that, as a result of drag encountered during half of the cycle, a symmetrical, rotational beating of node cilia is translated into a right to left fluid flow across the node [10]. Experimental reversal of nodal flow results in *situs* (laterality) reversal later in embryogenesis [11].

Differential gene expression is first seen in the crown cells at the periphery of the node itself. *Nodal* (a secreted morphogen of the TGFβ/BMP family) becomes expressed more strongly on the left than the right side [12,13] while expression of the *Nodal* inhibitor *Cerl2* (MGI: *Dand5*) shows the opposite distribution [14]. Both genes are initially expressed symmetrically before acquiring their asymmetric pattern. In the *iv* mutant, which lacks functional node cilia, asymmetric expression of *Cerl2* fails to become established [15] and right-sided expression of *Cerl2* has been shown to depend on an asymmetric left-sided flow-dependent post-transcriptional decay of *Cerl2* mRNA [16]. The mechanism by which this leftward fluid flow is transduced into differential gene expression is a matter of debate in the literature and a detailed discussion is beyond the scope of this review (for one see Norris [17]). One theory proposes that nodal crown cilia contain a mechanosensor, probably a complex consisting of the cation channels Pdk1l1 and Pdk2, leading to generation of a calcium wave in response to mechanical displacement [18,19]. A rival theory is that a morphogen may be secreted into the nodal pit and transported in the flow. Transport of Cerl2 has been demonstrated [15] although the timing suggests this occurs after the initial symmetry breaking event and seems to function to terminate *Nodal* expression. Transport of vesicles containing SHH and retinoic acid has also been demonstrated [20], and it is possible other morphogens may also be transported in this way.

Shortly after asymmetric expression of *Nodal* is seen in the node, it becomes expressed on the left side of the lateral plate mesoderm (LPM). *Nodal* expression in the node is a prerequisite for LPM gene expression [21] but whether or not Nodal is directly transported from the node to the LPM remains controversial. Such transport would most likely involve an interaction with sulphated glucosaminoglycans in the extracellular matrix of intervening tissue and inhibition of glucosaminoglycans inhibits *Nodal* expression in the LPM [22] but transport of Nodal has not been demonstrated. The gut endoderm appears to play a role in this relay. Gap junctional coupling has been demonstrated within the gut endoderm and this forms an interconnected network linking the node to the LPM on either side of the embryo, with the midline lacking gap junctions [23,24]. This network is disrupted in embryos lacking the transcription factor *Sox17* and a lack of gap junctional coupling in these embryos has been proposed to explain their failure to upregulate the *Nodal-Pitx2c* pathway in the LPM [23,24]. The nature of the signal transferred through these gap junctions remains to be established but the calcium wave generated at the node has to be a strong candidate.

Through activation of its receptor complex (consisting of Cfc1, Acvr1b and Acvr2b) and subsequent downstream signalling via Smad2/3/4 and the co-factor Foxh1, Nodal is able to activate its own transcription [25] as well as that of the transcription factor *Pitx2c* [26]. The function of Nodal is one of amplification through positive feedback, transforming a weak initial stimulus into a robust Left signal. As Nodal also activates transcription of its own repressors—*Lefty1* at the midline and *Lefty2* in the LPM [27]—negative feedback serves to limit transmission of the signal, restraining it to one side of the lateral plate.

Once activated, *Pitx2c* expression is also amplified through positive feedback but is not constrained by repression and expression persists in left-derived mesoderm structures throughout development. Pitx2c has been shown to be the major Left determinant in structures such as the atria and lungs [28].

### 2.2 Axis Establishment in the Chick Embryo

While the core left-sided *Nodal-Pitx2* signalling pathway is conserved in the chick [29,30] there are many important differences in left-right specification from mouse embryos (Figure 1). As in the mouse, asymmetric gene expression is first seen around the node before later being seen in the LPM. However, whereas only a small number of genes show asymmetric perinodal expression in the mouse, a large number—at least 15—have been described in the chick and most of these genes are expressed symmetrically in the mouse [31]. Both a left and a right-sided network of gene regulation is established at the chick node. On the left of the node, unlike in the mouse, *Shh* is asymmetrically expressed and this morphogen is required to transfer signals from the node to the left LPM where it activates *Nodal* expression [32,33]. *Fgf8* is expressed on the right side of the node and seems to function in repressing expression of *Nodal* in the right LPM [34]. Further lateral inhibition occurs within the LPM in which *Nodal* represses expression of the transcription factor *Snai1* in the left LPM while *Snai1* represses *Pitx2* expression in the right LPM [35]. The chick orthologue of *Cerl2*, *Cerberus/Caronte*, is expressed in the left LPM and not in the node, but there is disagreement in the literature as to whether it functions to promote Nodal expression or acts within a negative feedback loop in a manner similar to that of mouse *Lefty2* [36–38]. The related gene *Dan* is expressed on the left side of the chick node [39].

The mechanism of breaking symmetry is also fundamentally different in the chick from that seen in the mouse. While a small number of cells in the node of the chick embryo (Hensen’s node) do appear to be monociliated, the frequency of these cells does not differ from that of the surrounding epithelium, and as a result the chick embryo does not possess a ciliated pit analogous to the mouse node [40]. For this reason, a left-right fluid flow cannot be generated across the node and the chick must use a different mechanism to break symmetry. Morphological asymmetry at the node appears to precede asymmetric gene expression [41]. Furthermore, experiments in which the node is dissected out of the embryo and transplanted in an orientation 180° to its original position have demonstrated that left-right pattering of the node is determined by an extrinsic signal derived from the surrounding tissue [33]. Time lapse imaging of fluorescently labelled cells has demonstrated an anticlockwise, leftward movement of cells around Hensen’s node [42]. This movement appears to transform the initially symmetrical expression of *Fgf8* and *Shh* into an asymmetric perinodal pattern with *Fgf8* becoming localised to the right side and *Shh* to the left [42] (Figure 1). Pharmacological inhibition of either Rho kinase or myosin II may be used to prevent these cell movements and embryos treated in this way fail to develop asymmetric *Fgf8* or *Shh* expression [43]. Thus, in the chick it seems cell migration around the node, rather than fluid flow within it, is required to establish asymmetric gene expression.

These cell movements are preceded by the establishment of a potential difference between the two sides of the embryo across the primitive streak. The right side is hyperpolarised relative to the left by the pumping of K^+^ ions out of cells on the right side of the embryo by means of an H^+^/K^+^ ATPase pump [44]. Communication between the two sides of the embryo is required for correct left-right specification, perhaps to allow transmission of this electrical gradient, and this communication appears to be mediated by a network of Cx43 containing gap junctions in the ectoderm linking the left and right sides of the embryo, bypassing the node itself [45]. Blocking gap junctional communication either pharmacologically or using blocking antibodies results in symmetrical bilateral expression of *Nodal* and *Shh* [45] while pharmacological inhibition of the H^+^/K^+^ ATPase pump disrupts cell movement around the node [43].

### 2.3 The Evolution of Left-Right Axis Establishment

**Figure 2:**
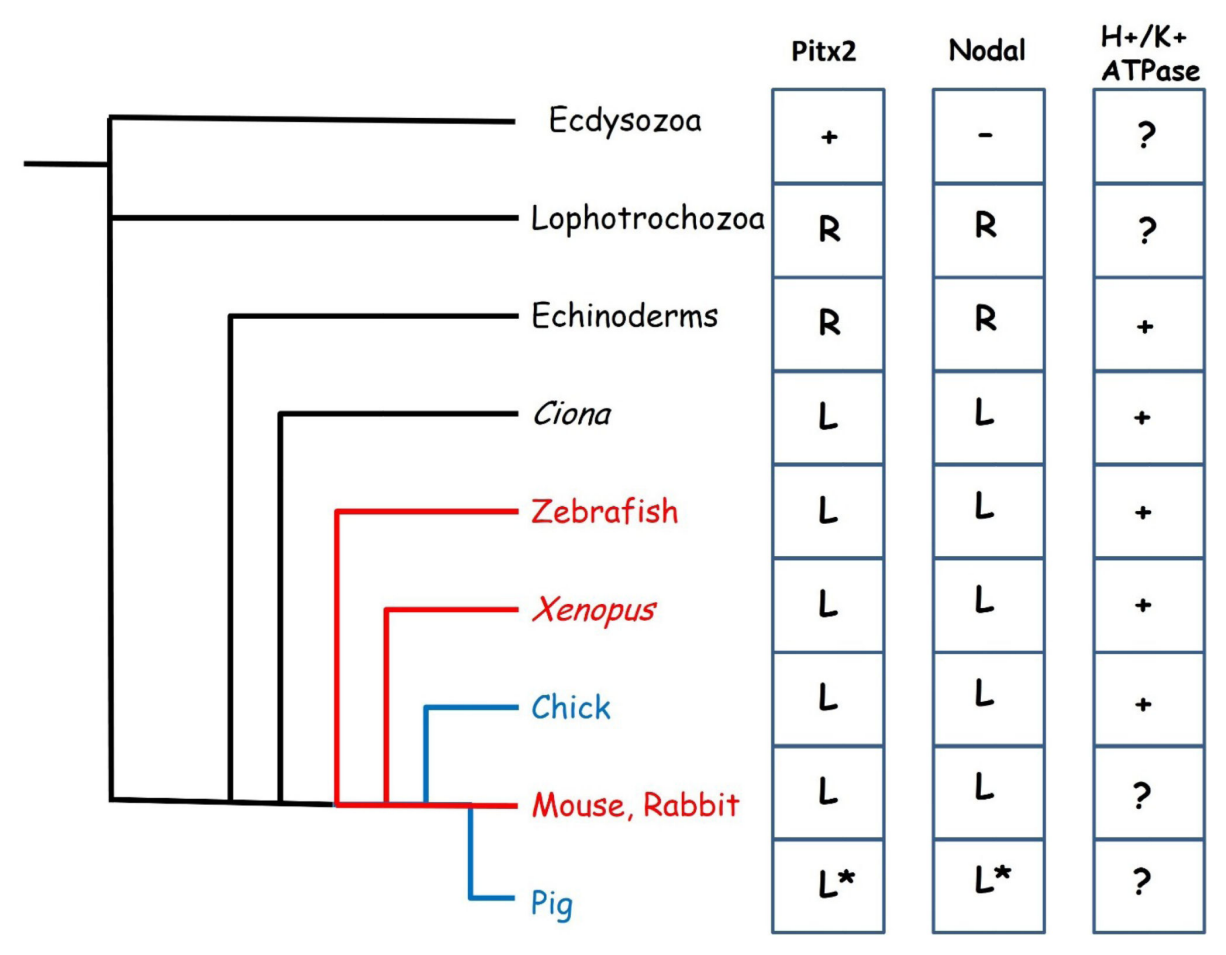
Evolution of left-right specification. The fluid flow mechanism of symmetry breaking is seen in many vertebrates (red lines) while cell movement based mechanisms have been described in the chick and pig (blue lines). To the right are shown the distribution of key left-right patterning genes across the animal kingdom. *Pitx2* is present in all animals shown but is not believed to play a role in left-right patterning in the Ecdysozoa which also lack *Nodal. Pitx2* and *Nodal* are expressed on the right side of the body in Lophotrochozoa and Echinoderms and left in all others. The expression of these genes has not been described in the pig, the * indicates assumed expression. A function in left-right specification for the H^+^/K^+^ ATPase has been described in Echinoderms, Tunicates and all vertebrates except mammals.

The symmetry breaking events in the mouse and chick embryo described above would seem to represent the two major mechanisms which exist in the animal kingdom. The fluid flow mechanism of symmetry breaking, in which a leftward flow is established across a ciliated pit as in the mouse is also seen in organiser regions of other embryos such as Kupffer’s vesicle of the zebrafish embryo [46] and in the gastrocoel roof plate, located in the dorsal endoderm of the archenteron of the *Xenopus* embryo [47,48] as well as that of other amphibians [49]. The node of the rabbit embryo, which develops as a flat disk similar to the human embryo, does not contain cilia but it has been shown that the adjacent posterior notochord plate has ciliated cells and a flow is generated here analogous to the nodal flow of other embryos [50]. Fluid flow is the most widespread symmetry breaking mechanism in the vertebrates (Figure 2) and has been hypothesised to be the ancestral vertebrate condition [49]. In contrast, the cell migration mechanism has been described in only two species, the chick embryo and the pig [43]. For a long time, the chick was the only embryo known to lack fluid flow, and being the only bird to have been studied, was assumed to represent a highly derived state [49]. The surprising discovery that the pig also uses this mechanism has cast doubt on this theory with the result that we can no longer state with confidence which of these mechanisms, if either, represents the ancestral state.

Convergence in developmental mechanisms seems to occur at the level of the *Nodal-Pitx2c* signalling pathway. The core components of the *Nodal-Pitx2c* pathway (that is to say, perinodal and LPM expressed *Nodal*, LPM expression of *Pitx2c* and *Lefty1* forming a midline barrier) are conserved across the vertebrates with minor variations [51]. This would appear to be a very ancient gene regulatory network whose origins may be traced much earlier in evolution (Figure 2) [52–54]. Homologues of *Pitx2* exist in *Drosophila* and *C.elegans* but do not seem to play a role in left-right patterning in these Ecdysozoans [55,56] which also lack a *Nodal* homologue [53]. *Nodal* appears to have evolved in a common ancestor of the Lophotrochozoa (Annelids and Molluscs) and the Deuterostomes and seems to have co-opted the pre-existing *Pitx2* gene into the left-right signalling pathway. Loss of either *Nodal* or *Pitx2* results in left-right patterning defects in both snails and in sea urchins [57,58]. In these animals, asymmetric expression is right-sided and it appears that a switch from right to left-sided expression occurred during evolution of the chordates, most likely as a result of the inversion of the dorsoventral orientation of the body plan [54]. Surprisingly, the *Nodal-Pitx2c* pathway does not seem to have evolved initially to pattern the mesoderm, but is expressed in various germ layers in different organisms [59].

The requirement for a potential difference generated by an H^+^/K^+^ ATPase pump for normal left right patterning is also conserved in many phyla, but has not been demonstrated in mammals (Figure 2), although it is not yet clear how this relates to the *Nodal-Pitx2c* pathway. Disruption of the H^+^/K^+^ ATPase in either *Xenopus*, zebrafish or chick leads to *situs* defects [44,60] indicating that this is an early mechanism of asymmetry conserved in phyla utilising both the cell migration and the fluid flow mechanisms of symmetry breaking. *Xenopus* embryos exhibit left-right asymmetry in expression of maternal mRNA from an early age, including that encoding the H^+^/K^+^ ATPase, which is localised as early as the 2 cell stage [61].

## 3 Cardiac Phenotypes Associated with Impaired Left-Right Patterning

The left-right axis is established in the lateral plate mesoderm prior to the major events in cardiogenesis. The primary and secondary heart fields are derived from lateral plate mesoderm as is the endothelium of the great vessels and these cells inherit positional information determined by the *Nodal-Pitx2c* pathway. It is therefore not surprising that many aspects of cardiac development are determined by patterning events established early in embryogenesis and that defective left-right patterning results in a range of cardiac phenotypes. A knowledge of both development and evolution can aide in the understanding of abnormalities seen in both human patients and in mouse mutants. In the second part of this review I will describe the major classes of cardiovascular abnormalities associated with defective LRP and discuss the underlying mechanisms. For a more detailed discussion, the reader is referred to an excellent review by Ramsdell [62].

### 3.1 Atrial Isomerism and Venous Drainage

**Figure 3:**
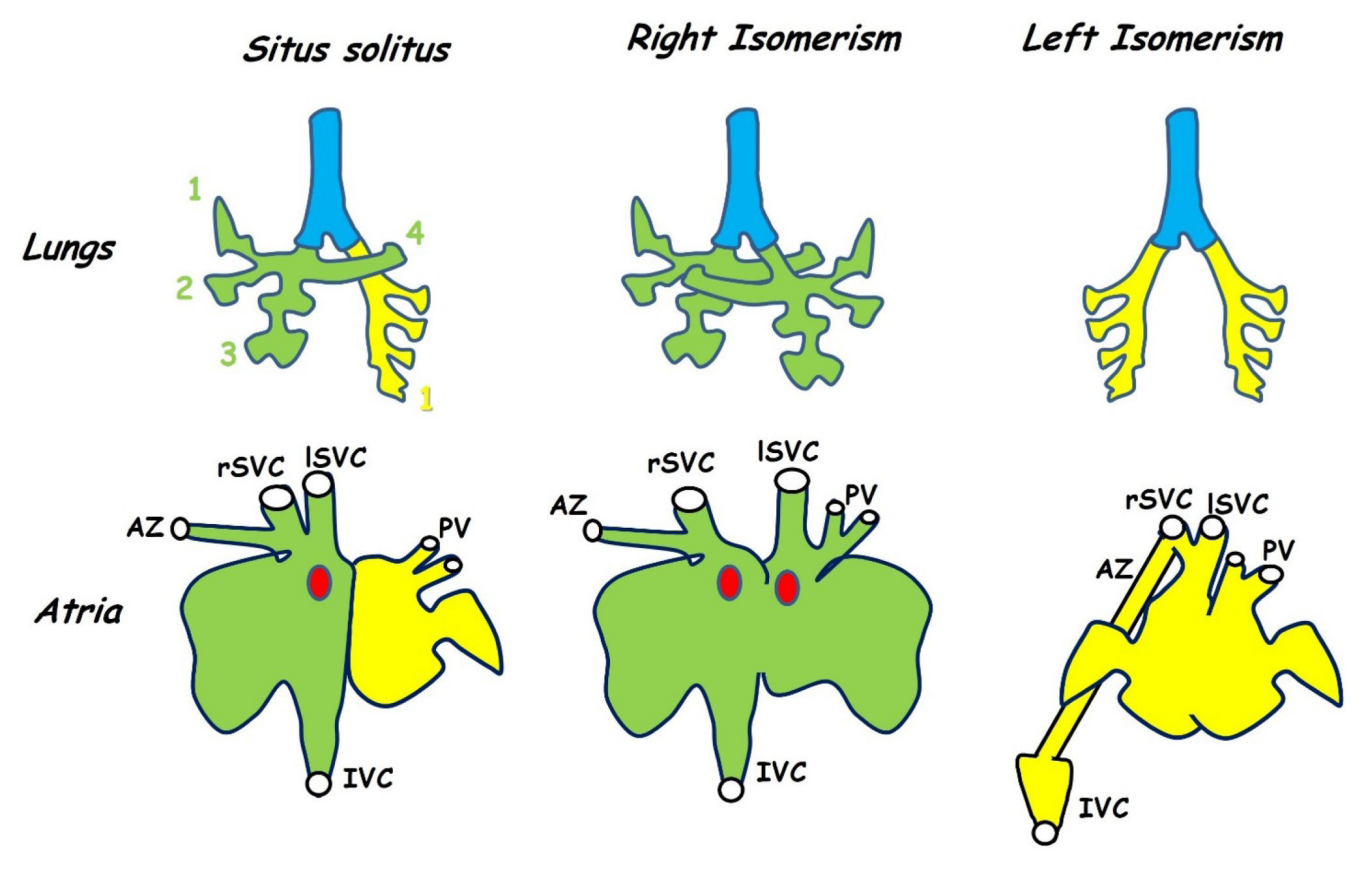
Isomerism is seen in both the lungs (**top row**) and in the atria (**bottom row**). This figure shows the anatomy of the mouse. *Situs solitus* is the normal morphology in which the right lung has four lobes (green) while the left has only one (yellow). Animals with left-right patterning defects may develop symmetrical lung morphology with either four lobes on each side (right isomerism) or one on each side (left isomerism). The atria differ both in morphology of the appendages (exaggerated in the figure for diagrammatic purposes) and in venous connections. In normal hearts both superior venae cavae (rSVC, lSVC) as well as the inferior vena cava (IVC) enter the right atrium while the pulmonary veins (PV) enter the left (*situs solitus*). The azygous vein (AZ) drains into the right superior vena cava. In hearts showing right isomerism the right SVC (rSVC) enters the right side while the left (lSVC) enters the left. The sino-atrial node (SAN, red dot) is duplicated and there is an atrial septal defect. Left isomerism is also associated with an atrial septal defect and both the venae cavae and pulmonary veins enter near the middle of a common atrium while the inferior vena cava is interrupted and drains into one of the superior venae cavae via the azygous vein. Variations are seen between the mouse and human phenotype (see text). Adapted from [63,67,68].

Isomerism represents one of the simplest forms of left right patterning defect and is seen in bilaterally paired organs of the thorax and abdomen such as the lungs which develop from initially symmetrical primordia. In the mouse a morphologically right lung is characterised by four lobes while a morphologically left lung has a single lobe, humans have a 3:2 lobed organisation [63]. Isomeric animals exhibit bilateral four or single lobed lungs, that is to say, morphologically the animal has two right lungs or two left lungs respectively (Figure 3) [64,65]. Pulmonary isomerism is usually, but not always, seen with atrial isomerism [66] and therefore provides an easily assayable indication of LRP defects, suggesting more complex cardiac defects may be present.

In the heart, isomerism is seen in the atria but not in the ventricles, this is because the atria develop from a common embryonic chamber that subsequently divides and differentiates asymmetrically while the two ventricles develop separately (Section 3.2). Left-right patterning defects result in both abnormal atrial morphology and in anomalous venous connections to the atria. In the mouse, the morphology of the i*v* mutant, which carries a mutation in the nodal cilia dynein *Dnah11* gene and therefore exhibits randomised *situs* (mice may be normal, mirror-image or isomeric) has been used as a frame of reference for this phenotype [67,68]. These studies have shown that the left and right atria may be distinguished on the basis of the morphology of the atrial appendages and that this is the most reliable indication of isomerism. For this reason, researchers often refer to isomerism of the atrial appendages rather than atrial isomerism itself (Figure 3) [64,67,69].

The venous system of the adult exhibits a high degree of asymmetry In addition to the asymmetry of left-sided pulmonary and right-sided systemic flow into the atria many veins are unpaired, such as the inferior vena cava and azygous. The adult venous circulation develops from the symmetrical arrangement seen in the early embryo by a process of asymmetric remodelling [70]. This process has parallels to the remodelling of the arterial system discussed below (Section 3.3). Remodelling is a complex process and the outcome of left-right patterning defects on this process is to some extent variable. Thus, while both atrial identity and the associated venous circulation are specified in part by left-right signalling, these two events occur independently and may be discordant [66,67,71].

The systemic input in mice consists of a pair of superior venae cavae and a single inferior vena cava, all three draining into the right atrium (Figure 3). In humans, a single superior vena cava enters the right atrium, blood from the left side of the head and upper body travels through the brachiocephalic vein to reach the right-sided superior vena cava. The pulmonary input consists of two pairs of pulmonary veins entering the left atrium.

Right isomerism in mice results in an anomalous symmetrical arrangement of the superior venae cava in which one vein enters each atrium or each side of a common atrium [72]. Bilateral superior venae cavae are also seen in human right isomerism cases, these result from a failure in the process of anastomosis between left and right anterior cardinal veins together with a failure in the normal regression of the proximal part of the right anterior cardinal vein [73]. Duplication of the inferior vena cava is not seen in right atrial isomerism and the single vein may enter either the right or the left atrium [74]. In some cases, hepatic veins may drain into the atria independently of the inferior vena cava [74]. Total anomalous pulmonary return, in which the pulmonary arteries do not enter the left-sided atrium, is seen in 97% of human cases with right atrial isomerism [66]. Variation is seen in the drainage site of these pulmonary veins but most commonly they enter the systemic circulation by draining into either of the superior venae cavae [66,74]. Anomalous pulmonary return does not seem to occur in *iv/iv* mice [72] but has been reported in the *Pitx2* null [65]. Persistence of a single umbilical vein draining into the common atrium has also been reported in mice lacking *Pitx2* [72].

Left isomerism is most commonly associated with interruption of the inferior vena cava which fails to drain into the right atrium and instead connects to the azygotic vein through which it drains into the superior vena cava [66,71,75]. This is also seen in *iv* mice with left atrial isomerism but is not thought to be a good indicator of left isomerism because it is also seen in a minority of animals with either normal or inverted *situs* [67]. A similar phenomenon has been reported in mice lacking *Lefty1*, which show left isomerism as a result of the lack of a midline barrier. In these mice, the inferior vena cava makes a normal connection to the right-sided atrium, but the azygous vein remains connected to it and thus forms a major conduit linking the inferior vena cava to the superior vena cava [64]. In these mice, the normally left-sided azygous vein becomes either right-sided or bilateral, additionally duplication of the inferior vena cava is frequent [64]. Bilateral systemic drainage is seen in left isomerism, for example in the *Shh* mutant, in which the superior venae cavae drain into a pouch-like confluence at the midline of the common atrium [69]. Partial anomalous pulmonary return is normally also a feature of left isomerism in humans. This is when a pair of pulmonary veins enter each atrium and thus half of the pulmonary circulation is misrouted [66,71,75]. Partial anomalous pulmonary return does not seem to occur in mice [64,67].

Because the right atrium contains the sino-atrial node and other specialised structures of the conduction system, right atrial isomerism is associated with cardiac arrhythmias as a result of node duplication [76] while left atrial isomerism is associated with hypoplastic or absent sino-atrial node [77]. *Pitx2c* expression is required in the left atrium during the late phase of asymmetric differentiation where it appears to actively suppress development of the conduction system [78,79].

### 3.2 Defective Looping Morphogenesis

**Figure 4:**
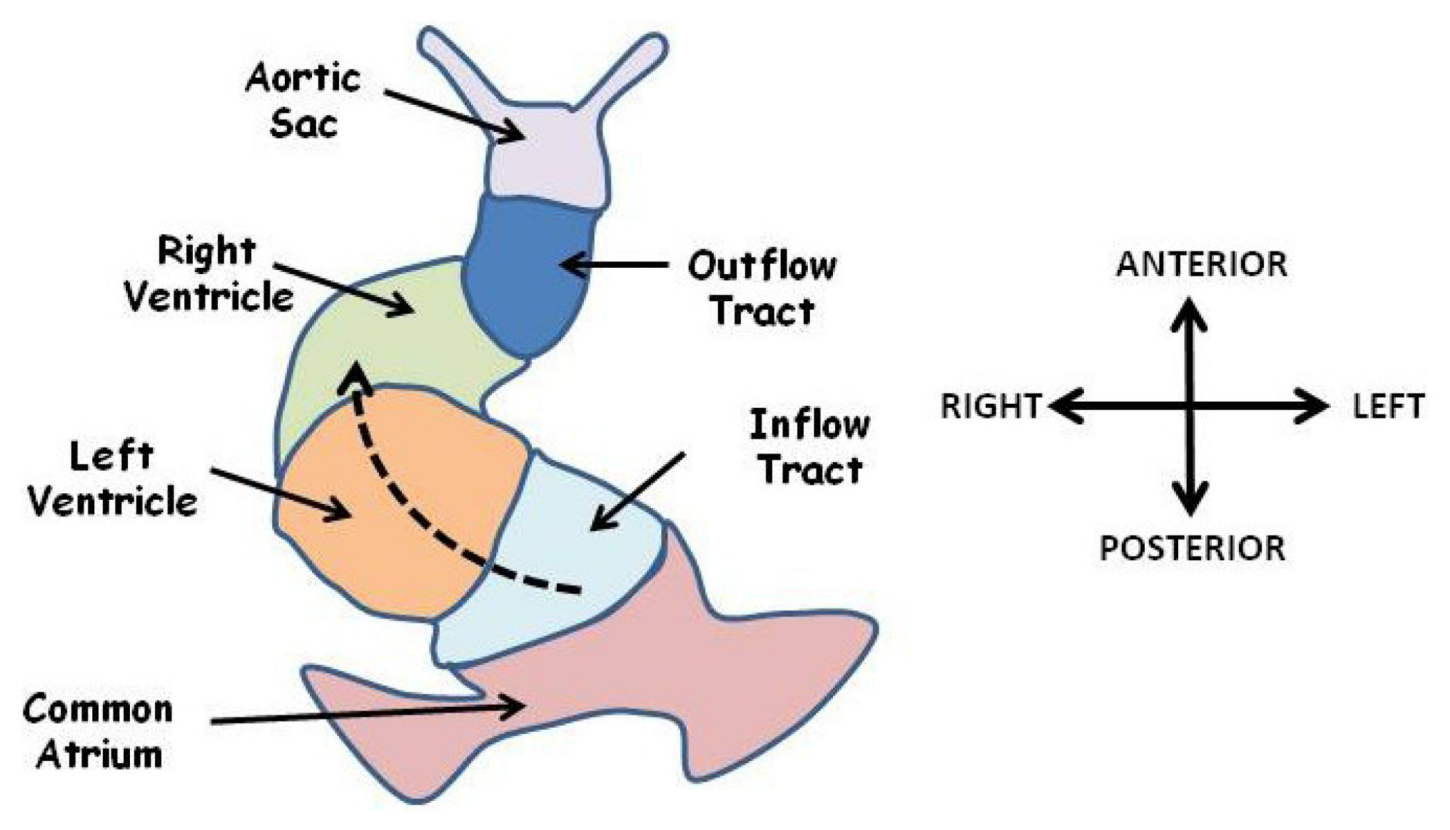
The linear heart tube is shown at the start of looping. The future ventricles lie in serial anterior to a common atrium. Blood enters a posteriorly located common atrium and flows in an anterior direction (dashed arrow) through the inflow tract, left ventricle, right ventricle, outflow tract and aortic sac. Looping morphogenesis will move the atrium anterior and rightward and precedes remodelling events to generate a four chambered, double circulation heart. Adapted from [81].

Early development of the heart is symmetrical resulting in a linear tube in which the future outflow tract, the right ventricle, left ventricle and common atrium are arranged in serial in an anterior to posterior sequence (Figure 4). This system acts as a simple pump with blood entering posteriorly and leaving anteriorly. Development of the mature four chambered double circulation heart of mammals and birds from this arrangement requires that the linear heart tube loops around itself in an asymmetric fashion, a process which occurs in two phases [5,80]. The first phase, often referred to as dextral looping, involves a rightward bulging of the mid portion of the heart tube (Figure 4) and results in formation of a helix with a single anticlockwise (left-handed) winding [81]. The second phase, often referred to as the S loop, results in a motif known as helical perversion in which the heart tube resembles two joined helices of opposite handedness [81]. These movements bring the two ventricles into apposition beside each other below the common atrium, and precede the extensive remodelling of the ventricular and atrioventricular septae. Looping also occurs in fish and other lower vertebrates to form a two-chambered heart [82]. In all vertebrates studied the heart normally loops to the right (as viewed in the direction of blood flow) [81] and looping morphogenesis is the first clearly visible break of symmetry in the embryo. Abnormal leftward looping results in a reversal in ventricle topology such that the morphological right ventricle (pulmonary circulation) is situated to the left of the heart and the morphological left ventricle (systemic circulation) on the right, examples in mouse include the *Zic3* knockout [83] and the *Cited2* knockout [84].

Looping is a complex process which appears to integrate signals from both the left-right axis and from the anterior-posterior axis. For example, looping fails to occur in mice lacking both the *HoxA* and *HoxB* gene clusters, which confer anterior-posterior identity [85]. In addition, looping morphogenesis is concurrent with the invasion of second heart field cells which populate the right but not the left ventricle, and looping does not occur in either the *Mef2c* or *Isl1* knockouts [86,87], both of which are expressed in the second heart field.

Ectopic over-expression of viral *Pitx2c* in the right LPM or antisense knockdown in the left LPM can randomise looping in the chick embryo [30,88,89]. Interestingly, many embryos with bilateral *Pitx2c* exhibit a symmetrical heart morphology with a bilateral bulging of the linear tube [30,88]. This result suggests that in the chick *Pitx2c* regulates flexing of the linear heart tube by changing the morphology of cells on one side. Support for this hypothesis comes from the observation that *Pitx2c* regulates asymmetric expression of the extracellular matrix protein Flexin [90]. Ectopic right-sided *Pitx2c* expression in *Xenopus* also leads to reversed looping [91].

In the mouse, *Pitx2c* loss of function does not result in reversed looping [72,92,93]. However, looping in mice does appear to be controlled in part by left-right patterning. *Nodal* loss of function mutants die during gastrulation before looping occurs but study of a hypomorphic allele has revealed that reducing the level of *Nodal* can randomise looping direction [94]. Similarly, mutations of the Nodal receptor *Cfc1* result in randomised looping [95]. Thus it would appear that in the mouse, directionality of looping is determined by a pathway downstream of *Nodal* but independent of *Pitx2c*. It is at present unclear why *Pitx2c* does not play a role in looping morphogenesis in the mouse.

### 3.3 Aortic Arch Defects

**Figure 5:**
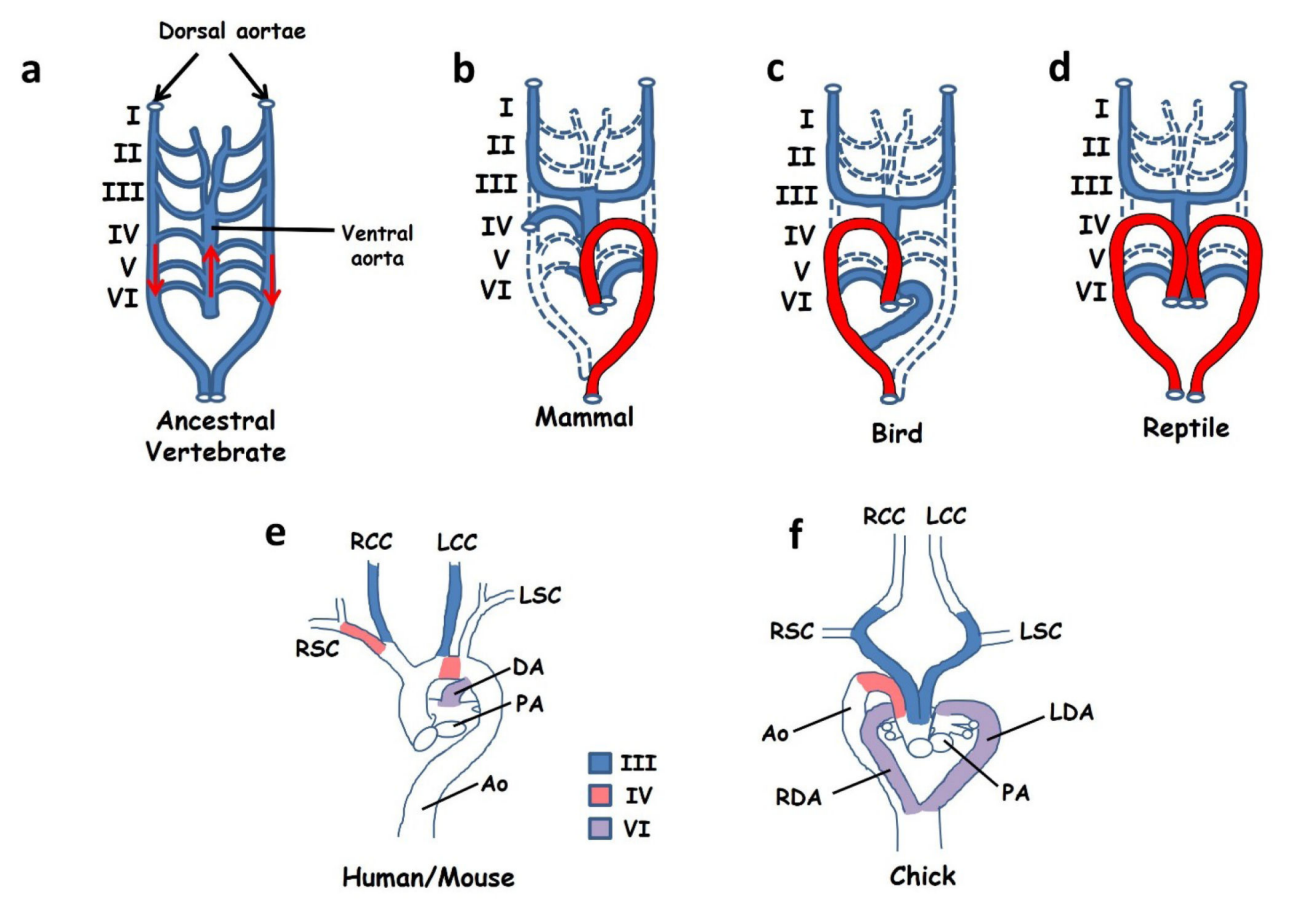
(**a**–**d**) Simplified cartoons to illustrate remodelling of the arterial circulation during embryogenesis. Ancestral vertebrates and modern day fish (**a**) use a symmetrical arterial system in which blood leaves the heart through a single ascending ventral aorta before entering a series of six paired arches (blood flow is indicated by red arrows). These vessels feed into a pair of descending dorsal aortae that carry oxygenated blood to the viscera. A similar circulation system is seen in early-mid stage embryos of all vertebrates. Remodelling during late embryogenesis involves regression of some vessels (dashed lines) with retention and expansion of others. Different remodelling strategies lead to a leftward looped aortic arch (shown in red) in mammals (**b**), a right arch in birds (**c**) and a double aorta in reptiles (**d**). Images in (**e**) and (**f**) show the anatomy of the great vessels in mice and humans at the time of birth (**e**) and in the chick at the time of hatching (**f**). The contributions of pharyngeal arch arteries III, IV and VI are indicated. (**a**–**d**) Adapted from [98] (**e**–**f**) adapted from [99]. Abbreviations: Ao: aorta, DA: ductus arteriosus, LCC: left common carotid, LDA: left ductus arteriosus, LSC: left subclavian, RCC: right common carotid, RDA: right ductus arteriosus, RSC: right subclavian.

Mammals have a leftward looped aortic arch in which the aorta exits the left ventricle, initially ascends and then loops to the left in order to descend into the thorax (Figure 5b,e). In contrast, birds have a rightward looped aortic arch (Figure 5c,f) while reptiles use a symmetrical circulation with one aorta on either side of the body (Figure 5d). The development of this system appears to be, in part, under control of the left-right signalling pathway and thus patterning defects may manifest in abnormal looping of the aortic arch. Mice with defective left-right patterning may exhibit a reversed, rightward looped aortic arch as well as, on occasion, a double aorta resembling that of reptiles [83,96,97]. In order to understand these defects we need to consider both the development and evolution of the great vessels.

The circulation system of all vertebrate embryos develops initially in a bilaterally symmetrical fashion in which a series of paired pharyngeal arch arteries leave the aortic sac and carry blood to paired descending dorsal aortae. The symmetrical circulation system of the early embryo is believed to be derived from that of a marine, gill-bearing vertebrate ancestor and is still seen in many teleosts. This ancestor is assumed to have had a symmetrical arterial tree consisting of a series of six paired branchial arch arteries carrying blood from a single ascending ventral aorta through the gills and into paired descending dorsal aortae carrying oxygenated blood to the viscera (Figure 5a) [98–100]. The pharyngeal arch arteries of both the mouse embryo (between days E9.5 and E10.5) and the chick embryo to some extent resemble this ancestral condition, consisting of an aortic sac (the ventral aorta), three symmetrical pairs of arch arteries (III, IV and VI) and two descending aortae [98,99]. The first and second pairs of arteries regress early in development while the fifth pair are transient in the chick and probably never seen in mice ([99] although see Bamforth *et al.* [101]).

Development of the mature aortic arch from the embryonic circulation requires extensive remodelling, a process in which some arteries regress into capillaries while others are retained. Remodelling is a highly asymmetric process and in many cases the developmental fates of each of a bilateral pair of arch arteries are quite different. In addition, differences in the left-right fates of these arteries between birds, mammals and reptiles leads to their differing adult morphology. In mammals the left IVth artery forms a part of the leftward looped aortic arch while the right becomes the proximal part of the right subclavian (Figure 5b,e) [99]. In contrast, in the chick the right IVth artery is maintained and becomes incorporated into the aorta while the left disappears (Figure 5c,f). In the case of the VIth artery, the distal portion of the right VIth regresses in mammals while the left is maintained until birth and becomes the ductus arteriosus linking the pulmonary and systemic circulations. This asymmetry is not seen in either birds or reptiles, both of which maintain both VIth arteries and develop two ductuses arteriosus [99,102]. Reptiles maintain a symmetrical arrangement in which one ductus feeds into each aorta, but in birds the left ductus must be routed into the single right aorta (Figure 5f).

The embryonic vascular system is formed by endothelium derived from the lateral plate mesoderm. In the fish, these precursors have been shown to express *Nkx2.5*, suggesting a common origin with the heart fields [103]. *Pitx2c* is expressed on the left side of the branchial arch mesoderm in mouse [96]. In the absence of *Pitx2c,* laterality of aortic arch looping is randomised: it may loop to the left or right or a double aorta may persist [96]. Mice lacking the Nodal receptor *Acvr2b* exhibit a similar phenotype [104]. Yet expression of *Pitx2c* on the left side of the LPM is conserved in vertebrates [105,106] suggesting either that it does not play a direct role in patterning the arteries or that interaction with other components of the pathway modifies its effect. We know that some left-right patterning genes show a reversed expression between mammals and birds. For example, FGF8 represses *Nodal* in the right LPM of the chick but is required for left LPM expression in mouse [107] while *Nkx3.2* expression in the chick LPM is a mirror image of that in the mouse [108]. Whether these early left-right events play a role in patterning the branchial arches is unclear. Arguing against such a role is the observation that remodelling of the arches is concurrent with invasion by neural crest cells which differentiate into vascular smooth muscle, and seem to be important in regulating persistence or degeneration of arteries [109]. *Cited2* mutant mice have a partially penetrant aortic arch defect together with abnormal neural crest migration through the arches [97]. Neural crest, being of ectodermal origin, is not patterned by the *Nodal-Pitx2c* pathway, but does migrate through LPM-derived tissue which could potentially influence its migration.

An alternative hypothesis is that the occurrence of such vascular defects in heterotaxic mutants is secondary to morphological changes in the heart itself. According to this hypothesis, remodelling of the vasculature is a biomechanical process and defective remodelling is driven by changes in blood flow resulting from changes in outflow tract morphology downstream of *Pitx2c* function in the second heart field [110].

## 4 Concluding Remarks: Left Right Patterning and Cardiac Disease

A number of congenital cardiac diseases have their origins in aberrant left right patterning, I have here discussed three examples from mouse genetics but there are many others. Cardiac looping is a pre-requisite for formation of the ventricular and atrioventricular septae, thus malformations of these structures may also in some cases be linked to defective left right patterning. Transposition of the great arteries shows a strong genetic association with asplenia (right isomerism) and thus rotation of the conotruncus is likely to be controlled in part by left-right patterning [111].

It is worth noting that left-right determination does not have to be an all or nothing affair: *Pitx2c* appears to act in a dose dependent manner, with reduced levels having been shown to affect some organ systems more severely than others [65,92]. This is an important observation, because in most cases human disease results not from gene loss of function but from heterozygosity. Heterozygosity for the left right patterning gene *Cited2* reduces but does not eliminate *Pitx2c* expression and is associated with a partially penetrant phenotype of ventricular septal defect and double outlet right ventricle [112]. Thus such forms of congenital heart disease, which are not clearly linked causally to left-right patterning, may in some cases nevertheless result from a weaker Left identity in parts of the heart.
